# Improving the Quality of Dental Laboratory Prescription Forms Through Training and Digital Workflow

**DOI:** 10.1155/ijod/8504529

**Published:** 2026-08-12

**Authors:** Shivani Kohli, Tuan Muhammad Asyraf, Muhammad Afiff, Nafij Bin Jamayet, Jagjit Singh Dhaliwal

**Affiliations:** ^1^ School of Medicine and Dentistry, Griffith University, Gold Coast, Australia, griffith.edu.au; ^2^ School of Dentistry, IMU University, Kuala Lumpur, Malaysia, imu.edu.my; ^3^ Division of Restorative Dentistry, School of Dentistry, IMU University, Kuala Lumpur, Malaysia, imu.edu.my

**Keywords:** dental education, digital innovation, lab form, paper-based, training

## Abstract

**Introduction:**

Communication between dentists and dental technicians traditionally depends on handwritten lab prescription forms. This method often results in issues like illegible handwriting, missing data, or lost forms, particularly among dental students. A digital platform was developed to address these challenges and streamline the transfer of patient information and preserve lab records. This study aimed to evaluate the impact of structured training on prosthodontic lab form completion and to assess the effectiveness of a newly developed digital lab form in enhancing communication and workflow between dental students and technicians.

**Methods:**

Structured training sessions were delivered to year 4 and year 5 dentistry students, involving practical exercises with mock patient records to complete paper‐based lab forms. Students’ feedback contributed to the creation of standardized form completion guidelines. A total of 156 lab forms (before and after training) were evaluated against a set of predetermined criteria by two independent examiners. Based on these criteria, a digital web application was developed, and year 5 students were introduced to the digital platform through a workshop and asked to complete forms for two sample prosthodontic cases. These digital submissions were assessed using the same criteria. Three datasets were analyzed: (1) quality of paper forms pre‐ and posttraining, (2) comparison of posttraining paper vs. digital forms, and (3) frequency of completed criteria. An email survey also collected student feedback on the training and digital platform. This mixed‐methods study combined quantitative assessment of the quality of laboratory prescription forms before and after training with qualitative feedback obtained from students and dental laboratory technicians regarding their experiences with the digital laboratory prescription platform.

**Results:**

Posttraining paper‐based forms improved significantly (mean score 11.12, standard deviation [SD] 1.35) compared to pretraining forms (mean 9.29, SD 1.49; *p*  < 0.01). Digital forms showed the highest quality (mean 13.00, SD 0.00) and outperformed year 4 posttraining paper forms (mean 10.84, SD 1.43; *p*  < 0.001). Digital forms also had significantly higher completion rates for required fields (*p*  < 0.01). Survey responses indicated the digital platform reduced errors, improved completeness, and strengthened communication with lab technicians. Training helped students identify common omissions and improved their understanding of lab form requirements.

**Conclusion:**

The digital lab form significantly enhanced the accuracy and completeness of prosthodontic prescriptions. Mandatory fields helped prevent incomplete submissions. The platform shows strong potential to improve clinic‐laboratory communication and workflow in dental education and practice.

**Clinical Relevance**

Effective communication between dentists and technicians is essential for accurate prosthodontic outcomes. Traditionally reliant on handwritten lab forms, this process is prone to errors such as omissions and illegibility, especially among dental students. While previous efforts focused on improving individual form completion skills, this study integrates structured training with digital innovation to address systemic issues. The introduction of a standardized digital lab form significantly improved data completeness and clarity. These findings suggest that incorporating digital tools and targeted training into dental education can enhance clinical communication, reduce errors, and ultimately improve the quality and efficiency of prosthodontic care in practice.

## 1. Introduction

Effective collaboration between dentistry students and dental technicians relies on a mutual understanding of each other’s roles, limitations, and responsibilities. As dentistry continues to grow rapidly, increased patient awareness and expectations have further emphasized the need for strong communication between dentists and technicians to achieve optimal clinical outcomes [1]. Several studies have consistently identified a persistent concern regarding inadequate communication and insufficient information provided by dentists when prescribing prostheses [2–10]. The dental laboratory prescription form remains the primary method of communication between dentists and technicians [3]. Clear communication of design specifications is essential, as it plays a critical role in the successful fabrication of both fixed and removable prostheses [6, 11, 12].

While some communication may occur by phone, the lab form is often the only written document guiding the technician’s work. Therefore, this form must include complete and accurate design instructions. The British Society for the Study of Prosthetic Dentistry emphasizes that it is the dentist’s responsibility to provide comprehensive design information and ensure the accurate transfer of all relevant details [13]. A well‐completed laboratory form contributes to the production of high‐quality prostheses, enhances patient satisfaction, and helps prevent errors or remakes—ultimately saving time and resources for both clinicians and technicians [14, 15].

The current dentistry curriculum does not include mandatory courses or workshops specifically focused on writing laboratory prescriptions. As a result, dentistry undergraduates typically acquire this skill only during their clinical years and often in a passive manner by filling out lab forms as cases arise rather than through structured training [16]. However, the habits and techniques students develop during clinical education often become ingrained and are carried into their future practice [17]. This underscores the need to introduce dedicated, practical training, such as intensive workshops or formal coursework, on writing accurate and comprehensive laboratory prescriptions. Multiple studies have emphasized the importance of effective communication with dental technicians during undergraduate clinical education [18–21].

To support this, the importance of correctly completing lab prescriptions should be reinforced from the early stages of dental education and consistently throughout. Reviewing these forms enables the identification of gaps in clinical practice relative to established standards, facilitating targeted improvements that enhance both service delivery and patient outcomes. Recognizing and addressing errors is integral to the pursuit of clinical excellence [7, 8].

In addition, accurate recordkeeping is crucial, as patient records serve not only clinical functions but also legal and administrative ones. The saying “good records facilitate good defence, poor records a poor defence, and no records no defence” reflects the significance of maintaining complete documentation [9].

The use of traditional, paper‐based forms to transfer patient information presents several challenges, including the risk of misplacement, illegible handwriting, and unclear diagrams—all of which can compromise the quality of communication between students and technicians [4, 10]. These inefficiencies often result in delays or require the forms to be rewritten entirely.

To address these challenges, the development of a secure, web‐based platform for transferring patient information to dental laboratories represents a promising solution, with the potential to improve accuracy, enhance communication, and reduce errors associated with traditional paper‐based systems. A digital system not only streamlines the workflow between clinics and laboratories but also helps ensure the integrity and accessibility of laboratory records.

## 2. Methods

This research project was approved by the 228th IMU Joint Committee on Research and Ethics (Project ID: BDS I‐01/2021(11)) and was conducted from August 2021 to June 2022.

Before the commencement of the research, all participants were provided with a participant information sheet (PIS) outlining the details of the study. Written informed consent was obtained from all participants before the project began. Participation in the workshops was voluntary, with 39 students from each year level taking part.

During the dental laboratory prescription training, communication between students and laboratory technicians was primarily via paper‐based lab forms. Common issues identified with these forms included incomplete patient information, insufficient prosthesis details, and illegible handwriting—all of which pose risks to the quality of patient care [3]. To address these challenges, structured training workshops focusing on dental laboratory prescription writing were conducted separately for year 4 and year 5 undergraduate dentistry students to improve the accuracy, completeness, and clarity of laboratory communication.

Prior to the workshops, two research students developed a set of criteria and standards for high‐quality lab prescription writing under the guidance of their research supervisors. These students were subsequently trained to assess the lab forms using these criteria. To ensure consistency in evaluation, an interobserver reliability test was conducted using Cohen’s kappa statistic. A final consensus was reached when the kappa value indicated an almost perfect agreement (0.81–1.0).

The student‐led workshop was conducted separately for each cohort. At the start of the session, all participating students were asked to complete paper‐based laboratory prescription forms for two sample prosthodontic cases. These forms were then collected by the research students for evaluation. Following this, the workshop focused on educating participants about the importance of a well‐written laboratory prescription in the dental profession. The criteria and standards developed for high‐quality lab prescriptions were thoroughly discussed and explained.

To evaluate the quality of the prescriptions written by students, a simplified assessment tool comprising 13 key criteria was used. This simplified version was derived from the comprehensive criteria outlined below.

The criteria to assess the quality of the dental lab form are as follows:

• Student’s name
• Supervisor’s name
• Patient’s name
• Patient’s age
• Patient’s gender
• Patient’s registration number
• Date sent to the lab
• Payment receipt number
• Type of prosthesis
• Shade of teeth
• Instructions written
• Date required
• Design drawn and indicated

The research students analyzed all collected lab forms for quality, using the preestablished guidelines. Each record was scored based on whether the criteria were met (scored as 1) or not met (scored as 0). The average score for each record across all criteria was calculated. Following this analysis, the finalized criteria were compiled and used as a guideline for students. These guidelines were then disseminated through class meetings, where all students were taught how to apply them effectively.

A new digital platform, a web‐based software application, was developed to facilitate the transfer of patient information for prosthodontic cases to the dental laboratory. The digital laboratory prescription form was designed and developed by Brand You Sdn Bhd and hosted under the registered domain https://digitaldentallabform.com. The platform was maintained on a paid domain and was active throughout the study period (August 2021–June 2022); it is currently inactive following completion of the project. As such, the platform functioned as a project‐specific prototype, and its outcomes are being used to inform recommendations for future institutional implementation.

The platform was designed based on established criteria for evaluating the quality of dental laboratory prescriptions. A workshop was conducted for all fifth‐year dental students, during which they were introduced to the digital laboratory form and trained in its use. Participants were required to complete the digital form for two sample prosthodontic cases. All submitted records were stored within the platform’s database, enabling dental laboratory technicians to access and review the prescriptions through the same system.

Following the training intervention, year 4 students continued using paper‐based lab prescription forms in clinical practice, whereas year 5 students transitioned to using the digital form for their clinical patient cases, enabling a comparative evaluation of traditional and digital workflows. After 3 months of practice [22], the quality of laboratory prescriptions was reassessed. Two paper‐based lab forms were selected from each year 4 student (totaling 78 forms), and two digital lab forms were selected from each year 5 student (totaling 78 forms). The forms were evaluated based on the criteria and standards developed earlier in the study.

All data were collected, analyzed, and tabulated using the statistical software SPSS version 25. Quantitative analysis was conducted to assess how many students accurately filled in all the required details in the digital lab form. Descriptive statistics were used to summarize frequencies, percentages, means, and standard deviations (SDs). Domain‐level comparisons were performed using the statistical procedures available. Paired *t*‐tests were used to compare pre‐ and posttraining laboratory prescription scores within the same students. Comparisons between year 4 students using paper‐based forms and year 5 students using digital forms involved independent cohorts and were analyzed as between‐group comparisons. Chi‐square tests were used to assess differences in the frequency of individual criteria recorded before and after training. Comparisons between year 4 students using paper‐based forms and year 5 students using digital forms were performed as between‐group analyses. Statistical significance was set at *p*  < 0.05.

For qualitative analysis, feedback was gathered from participants via email‐based surveys using Likert scale items to capture their levels of agreement with key aspects of the intervention. Participants were asked about their satisfaction with the training on writing lab forms and using the digital lab form. They were also invited to provide comments, recommendations, and suggestions for improvements. The following questions were posed to solicit qualitative responses:

1. How satisfied were you with the experience of using the digital lab form?
2. How relevant and helpful do you find the digital lab form compared to the paper‐based version?
3. Would you recommend using this digital platform for future clinical cases?
4. How satisfied were you with the training sessions?
5. Do you have any overall feedback on the digital lab form?

Feedback from dental technicians was also collected to gather their opinions, comments, and suggestions for improving the functionality of the digital lab form and enhancing its convenience.

## 3. Result

### 3.1. Quantitative Analysis

The set of criteria consisted of eight major components, each with its minor subcomponents. These criteria were used to assess how frequently students filled out the lab prescription forms. Some criteria were scored as 0 because the existing paper‐based lab form did not include the corresponding sections for students to complete. Other criteria, particularly those related to the types of prostheses prescribed, showed lower frequencies as students often chose to prescribe only one type of prosthesis at a time. Additionally, some sections did not apply to every student. A total of 78 forms were collected and analyzed for both pretraining and posttraining periods. The number and percentage of records that met each criterion before and after training are shown in Table 1.

**Table 1:** Frequency of criteria written in the paper‐based lab form pre‐ and posttraining.

Criteria	Pretraining (%)	Posttraining (%)	*p*‐Value
Student details:			
Name	66 (84.61%)	78 (100%)	<0.001
Student’s ID	0	0	0
Cohort	0	0	0
Supervisor’s name	65 (83.33%)	69 (88.46%)	0.245
Patient’s detail:			
Name	78 (100%)	78 (100%)	—
Age	78 (100%)	78 (100%)	—
Gender	78 (100%)	78 (100%)	—
Registration number (RN)	78 (100%)	78 (100%)	—
Technician’s details:			
Name	58 (74.36%)	63 (80.77%)	0.221
Lab record number	58 (74.36%)	63 (80.77%)	0.221
Lab details:			
Type of prosthesis	76 (97.44%)	78 (100%)	0.248
Date sent to the lab	51 (65.38%)	45 (57.69%)	0.205
Date of request	24 (30.77%)	24 (30.77%)	0.569
Clinic stamp	47 (60.26%)	45 (57.69%)	0.435
Payment receipt number	38 (48.72%)	59 (75.64%)	<0.001
Supervisor signature	63 (80.77%)	52 (66.67%)	0.340
Technician’s signature	32 (41.02%)	29 (37.18%)	0.371
Removable prosthesis:			
Type of prosthesis	42 (91.30%)	56 (96.55%)	0.236
Arch indicated	44 (95.65%)	56 (96.55%)	0.599
Teeth to be replaced	36 (78.26%)	37 (63.79%)	0.082
Type of material	42 (91.30%)	57 (98.26%)	0.118
Shade of teeth	29 (63.04%)	52 (89.66%)	0.001
Shade guide	0	4 (6.90%)	0.092
Diagrammatic label	21 (45.65%)	39 (67.24%)	0.022
Cobalt chrome:			
Type of major connector	16 (59.26%)	26 (72.22%)	0.209
Type of clasp assembly	4 (14.82%)	20 (55.56%)	<0.001
Location of clasp	8 (29.63%)	32 (88.89%)	<0.001
Location of abutment	8 (29.63%)	29 (80.56%)	<0.001
Type of rest	0	10 (27.78%)	0.002
Location of rest	11 (40.74%)	27 (75.00%)	<0.001
Guiding plane	0	7 (19.44%)	0.015
Fixed prosthesis:			
Type of prosthesis	29 (100%)	21 (100%)	—
Type of alloy used	21 (72.41%)	16 (76.19%)	0.514
Diagrammatic indication	17 (58.62%)	14 (66.67%)	0.390
Shade of teeth	23 (79.31%)	19 (90.48%)	0.255
Type of shade guide	3 (10.34%)	5 (23.80%)	0.186
Tooth number	29 (100%)	21 (100%)	—
Type of material	25 (86.20%)	19 (90.48%)	0.501
All ceramic crown	0	0	0
PFM crown design	3 (10.34%)	1 (4.76%)	0.436
Pontic design	1 (3.45%)	2 (9.52%)	0.379
Tooth to be replaced	11 (37.93%)	11 (52.38%)	0.233
Feedback of the prosthesis:			
Satisfactory score	33 (42.31%)	30 (38.46%)	0.372
Comment	10 (12.82%)	6 (7.69%)	0.215

For the student details section, statistically significant improvements were observed for the recording of student names. However, no statistically significant differences were found in the recording of supervisor names between pre‐ and posttraining. All patient‐related domains were completely documented in both pre‐ and posttraining forms. Documentation of technician details showed only marginal improvements in frequency and percentage, without statistical significance. Of the laboratory‐related variables, only the payment receipt number exhibited a statistically significant improvement. While other criteria showed variations, the differences between pre‐ and posttraining were not statistically significant.

In the removable prosthesis section, both tooth shade and diagrammatic labeling showed statistically significant improvements. For the cobalt chrome section, all criteria, except for the type of major connector, showed statistically significant improvements between pre‐ and posttraining. In the fixed prosthesis section, both the type of prosthesis and tooth numbers were fully recorded in both pre‐ and posttraining forms. However, the remaining criteria showed some variations, but the differences were not statistically significant.

Table 2 presents the mean scores of pre‐ and posttraining criteria met by year 4 and year 5 students. The mean total score for posttraining (11.1220 ± 1.34527) was significantly higher than the mean score for pretraining (9.2927 ± 1.48734), with a *p*‐value < 0.01.

**Table 2:** Quality of written lab forms pretraining and posttraining.

Mean (SD)				
Variables	Pretraining	Posttraining	*t*‐Statistic (df)	*p*‐Value
Total scores	9.2927 (1.48734)	11.1220 (1.34527)	5.267(40)	<0.01

Table 3 compares the mean scores for year 4 students posttraining with paper‐based forms and year 5 students posttraining with digital forms. The mean total score for year 5 (13.00 ± 0.00) using the digital platform was significantly higher than the mean score for year 4 (10.8409 ± 1.42964) using paper‐based forms, with a *p*‐value < 0.001.

**Table 3:** Quality of lab forms posttraining year 4 using paper‐based and posttraining year 5 using digital‐based.

Mean (SD)				
Variables	Year 4 posttraining paper‐based	Year 5 posttraining digital	*t*‐Statistic (df)	*p*‐Value
Total scores	10.8409 (1.42964)	13.000 (0.000)	10.469 (90)	<0.001

Table 4 shows the mean number of criteria met within each domain, both pre‐ and posttraining. Statistically significant improvements were noted in three major domains for posttraining data. Table 5 summarizes the mean number of criteria met and the SD for each domain in year 4 and year 5, both pre‐ and posttraining.

**Table 4:** Mean number of criteria met and standard deviation (SD) for each domain pre‐ and posttraining.

Number of criteria met	Pretraining (*n* [SD])	Posttraining (*n* [SD])	*p*‐Value (<0.05)
Student’s details (*n* = 4)	1.615 (0.747)	1.897 (0.307)	0.032
Patient’s details (*n* = 4)	3.974 (0.160)	4.00 (0.00)	0.32
Technician’s details (*n* = 2)	1.231 (0.986)	2.00 (0.00)	0.001
Lab details (*n* = 7)	4.410 (1.534)	4.128 (1.281)	0.381
Removable prosthesis (*n* = 7)	4.645 (1.050)	3.10 (1.185)	0.118
Cobalt chrome (*n* = 7)	2.00 (1.782)	3.778 (1.592)	0.003
Fixed prosthesis (*n* = 11)	6.00 (1.414)	6.778 (1.716)	0.349
Feedback (*n* = 2)	0.205 (0.409)	0.359 (0.584)	0.182

**Table 5:** Mean number of criteria met and standard deviation (SD) for each domain in year 4 and year 5, pre‐ and posttraining.

Number of criteria met	Year 4			Year 5		
	Pretraining (*n* [SD])	Posttraining (*n* [SD])	*p*‐Value (<0.05)	Pretraining (*n* [SD])	Posttraining (*n* [SD])	*p*‐Value (<0.05)
Student’s details (*n* = 4)	1.615 (0.747)	1.897 (0.307)	0.032	1.74 (0.549)	1.87 (0.339)	0.281
Patient’s details (*n* = 4)	3.974 (0.160)	4.00 (0.00)	0.32	3.82 (0.389)	3.92 (0.270)	0.18
Technician’s details (*n* = 2)	1.231 (0.986)	2.00 (0.00)	0.001	1.74 (0.677)	1.23 (0.986)	0.009
Lab details (*n* = 7)	4.410 (1.534)	4.128 (1.281)	0.381	4.08 (1.20)	4.38 (1.388)	0.298
Removable prosthesis (*n* = 7)	4.645 (1.050)	3.100 (1.185)	0.118	4.667 (0.976)	5.286 (0.937)	0.048
Cobalt chrome (*n* = 7)	2.00 (1.782)	3.778 (1.592)	0.003	1.222 (1.641)	4.889 (2.139)	0.001
Fixed prosthesis (*n* = 11)	6.00 (1.414)	6.778 (1.716)	0.349	5.611 (1.335)	5.667 (1.371)	0.913
Feedback (*n* = 2)	0.205 (0.409)	0.359 (0.584)	0.182	0.897 (0.754)	0.564 (0.680)	0.044

### 3.2. Qualitative Analysis

Out of the 20 participants, seven students provided qualitative feedback. Their responses were grouped into four key segments: student satisfaction with the training, satisfaction with the use of digital lab forms, opinions on the relevance of digital lab forms, and overall recommendations. Most students expressed satisfaction with both the training session and their experience using the digital lab form, with only one student rating their experience as average. This sentiment extended to their views on the relevance and helpfulness of the digital lab forms. Some responses included “The newly developed digital lab form is useful,” “It’s more convenient than paper‐based forms as changes can be easily made after submission,” and “It eliminates the need to refill a new paper‐based lab form.”

Feedback from dental lab technicians was also gathered. They noted that “The digital form is clear, easy to use, and well‐detailed” and “I think we could make a few sections mandatory so users cannot save the form without filling them in—great job! I didn’t expect the drawing feature, good job!” However, one technician mentioned, “There’s too much information to enter. For the patient details section, it would be enough to include just the doctor’s name, dental technologist, patient name, age, gender, RN number, lab number, and receipt number.”

## 4. Discussion

Dentistry students are expected to develop a wide range of skills in prosthodontics, including both clinical and laboratory techniques. However, poor communication, particularly between dentists and dental technicians, remains a longstanding issue [17]. Numerous studies worldwide have highlighted the poor transfer of prosthodontic data between dentists and dental technicians [4, 8]. The purpose of this research was to assess the impact of incorporating lab prescription training into the undergraduate curriculum and the effectiveness of a newly developed digital laboratory form. This study is unique in that it involves a student‐led training approach with active participation from other students.

The findings of this study demonstrate that dental laboratory prescription training, combined with the use of a newly developed digital prescription form, can positively influence clinical practice. Significant improvements were observed across several criteria completed by students following the training, particularly in the accuracy of prosthodontic prescription documentation [22]. This improvement was seen in both year 4 and year 5 students, suggesting that lab prescription training can enhance the accuracy of form completion across different stages of dental education. Additionally, the study compared results from students in different years, focusing on the completeness of the required criteria.

Similar to findings by Alammari and Albagar [17] and Stewart [23], this study revealed that in 69.23% of cases, the date when the prosthesis was required was missing and not filled in the designated area of the lab form. Furthermore, 33.33% of cases lacked the diagrammatic design or indication for removable prostheses. These issues may stem from challenges dentistry students face in designing prostheses, as well as time constraints in the clinic due to poor time management or the complexity of the procedures involved [17]. As emphasized in previous studies, the technician’s role is to fabricate the prosthesis according to the specifications provided in the prescription [2, 3]. If these details are missing or unclear, the technician is left to make critical decisions, which can lead to undesirable outcomes. Thus, the success of prosthesis fabrication heavily depends on clear and effective communication between the dentist and the technician. Miscommunications in the prescription process can significantly affect the quality of the final prosthesis [23].

Through training, greater emphasis should be placed on ensuring that laboratory prescription forms are completed accurately and thoroughly. Courses led by prosthodontists and dental technicians are crucial for enhancing communication between dentists and technicians [17]. Dickie et al. [20] reported that after providing additional information on this topic, including incorporating prescription‐writing questions into undergraduate objective structured clinical examinations, they observed improvements in prescription and form completeness. One effective approach to encourage students to complete prescriptions accurately is to simplify the process and include extensive guidelines and model examples for both removable and fixed prosthodontic cases [17, 24, 25]. Similar results were observed in this study, where training in lab form writing led to a significant improvement in the quality of prescriptions sent to dental technicians (*p*  < 0.01).

**Figure 1:**
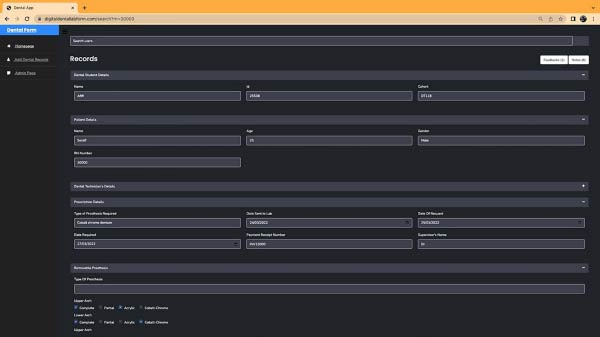
New dental records section.

**Figure 2:**
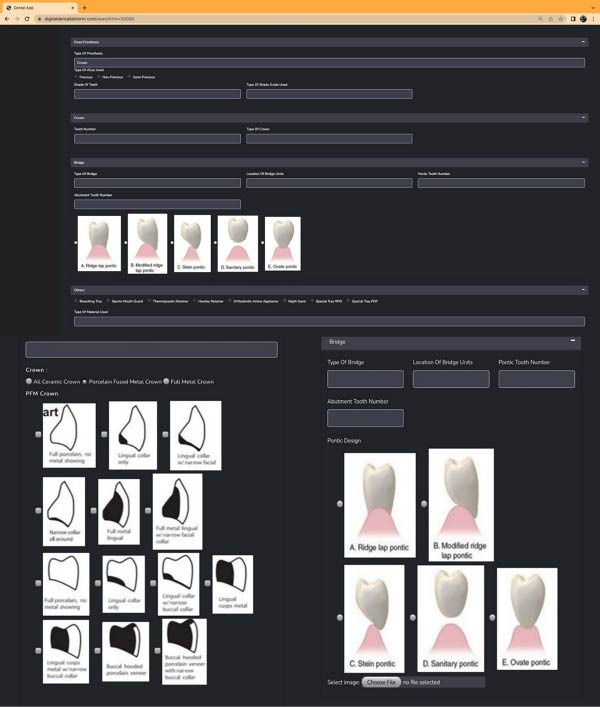
Fixed prosthesis section, PFM crown design, and pontic design selection.

**Figure 3:**
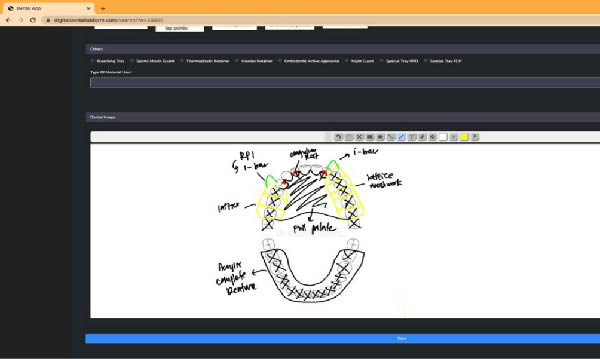
Prosthesis design section.

**Figure 4:**
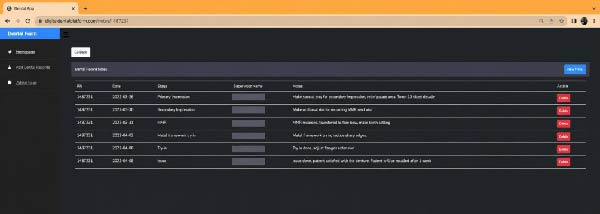
Example of instructions and stages of prosthesis.

**Figure 5:**
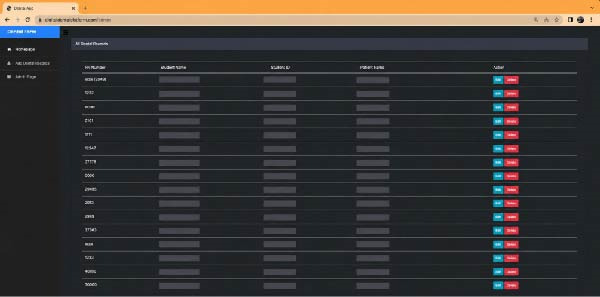
Admin page; list of saved dental lab records can be viewed by user.

The integration of electronic and digital technologies, not only in teaching and learning but also in everyday clinical practice, offers endless possibilities [26]. With digital tools, users can easily select options, view, edit, and delete records as necessary, allowing for real‐time updates during the prosthesis fabrication process. The use of the new digital lab form enabled students to provide all required details, ensuring that dental lab prescriptions were complete (Figure 1). It also incorporated fixed prosthesis options along with different crown and pontic designs (Figure 2). This reduces the likelihood of incomplete forms and enhances communication between dental practitioners and lab technicians [7, 9, 11]. Similar to Al‐Samawi and Mahmood [26], this digital platform includes a built‐in drawing section where students can draw, erase, and adjust prosthesis designs, thereby improving both accuracy and efficiency (Figure 3). Furthermore, the platform allows users to include detailed instructions for the technician, encompassing various stages of prosthesis fabrication (Figure 4). Numerous studies have highlighted the effectiveness of computer‐based platforms for designing removable partial dentures (RPDs) [27–29]. Furthermore, technicians can access and view these records through the digital interface (Figure 5).

The qualitative feedback from students further supported the quantitative findings. The survey was conducted online to increase engagement and convenience for respondents. Previous research has demonstrated that online and postal surveys yield similar response rates when specific protocols are followed. After the training, students reported feeling more confident in their ability to complete lab forms compared to their previous experience. A substantial proportion of respondents recommended that training in laboratory prescription forms, especially digital formats, be introduced earlier in the curriculum, preferably when students first commence clinical patient treatment. They also expressed that a digital lab form would be an invaluable tool in improving the quality of dentistry students’ lab forms.

## 5. Conclusion

The findings indicate a significant improvement in the quality of laboratory prescription forms following the training, as reflected by the substantial differences between pre‐ and posttraining scores among both year 4 and year 5 students. As the comparison between paper‐based and digital workflows involved different student cohorts (year 4 and year 5), the observed differences may have been influenced by cohort‐specific factors, including variations in clinical experience and baseline competency. With a *p*‐value < 0.05, we reject the null hypothesis, confirming that the implementation of training and the use of digital lab forms led to substantial improvements. Specifically, year 5 students, who used the newly developed digital platform, showed significantly higher adherence to the required criteria compared to year 4 students using traditional paper‐based forms. The digital platform’s features, such as making student and patient details mandatory before form submission, ensured that critical information was consistently included, reducing the risk of omissions. This enhanced accuracy and efficiency underscore the digital form’s potential to revolutionize communication between dental students and technicians, ultimately improving patient care outcomes.

However, the platform interface was developed as a functional prototype with an emphasis on usability, with opportunities for further enhancement through advanced visual design features. These improvements can be incorporated into future development to enhance user experience and better align the platform with clinical practice.

## Author Contributions

**Shivani Kohli**: writing – review and editing, conceptualization, resources, methodology, project administration, supervision, validation. **Tuan Muhammad Asyraf**: methodology, data curation, writing – original draft preparation, software, investigation. **Muhammad Afiff**: visualization, writing – original draft preparation, investigation, methodology, data curation, software. **Nafij Bin Jamayet**: methodology, writing – review and editing, project administration, supervision. **Jagjit Singh Dhaliwal**: resources, writing – review and editing, validation.

## Funding

This research was funded by the International Medical University (IMU) Joint Committee on Research and Ethics under Project ID Number BDS I‐01/2021(11). Open access publishing facilitated by Griffith University, as part of the Wiley ‐ Griffith University agreement via the Council of Australasian University Librarians.

## Disclosure

All authors have read and approved the final version of the manuscript. Shivani Kohli had full access to all of the data in this study and takes complete responsibility for the integrity of the data and the accuracy of the data analysis.

## Conflicts of Interest

The authors declare no conflicts of interest.

## Data Availability

The authors confirm that the data supporting the findings of this study are available within the article. The datasets used during the current study are available from the corresponding author upon reasonable request.
